# CEPICS: A Comparison and Evaluation Platform for Integration Methods in Cancer Subtyping

**DOI:** 10.3389/fgene.2019.00966

**Published:** 2019-10-08

**Authors:** Ran Duan, Lin Gao, Han Xu, Kuo Song, Yuxuan Hu, Hongda Wang, Yongqiang Dong, Chenxing Zhang, Songwei Jia

**Affiliations:** School of Computer Science and Technology, Xidian University, Xi’an, China

**Keywords:** data integration, multi-omics, cluster analysis, cancer subtypes, R package

## Abstract

Cancer subtypes can improve our understanding of cancer, and suggest more precise treatment for patients. Multi-omics molecular data can characterize cancers at different levels. Up to now, many computational methods that integrate multi-omics data for cancer subtyping have been proposed. However, there are no consistent criteria to evaluate the integration methods due to the lack of gold standards (e.g., the number of subtypes in a specific cancer). Since comprehensive evaluation and comparison between different methods serves as a useful tool or guideline for users to select an optimal method for their own purpose, we develop a scalable platform, CEPICS, for comprehensively evaluating and comparing multi-omics data integration methods in cancer subtyping. Given a user-specified maximum number of subtypes, *k*-max, CEPICS provides (1) cancer subtyping results using up to five built-in state-of-the-art integration methods under the number of subtypes from two to *k*-max, (2) a report including the evaluation of each user-selected method and comparisons across them using clustering performance metrics and clinical survival analysis, and (3) an overall analysis of subtyping results by different methods representing a robust cancer subtype prediction for samples. Furthermore, users can upload subtyping results of their own methods to compare with the built-in methods. CEPICS is implemented as an R package and is freely available at https://github.com/GaoLabXDU/CEPICS.

## Introduction

With the development of high-throughput technologies, huge amounts of multi-omics data for cancers have been generated, such as genomics, epigenomics, and transcriptomics data. Researchers have opportunities to integrate different types of omics data to advance cancer research, including identifying cancer driver mutations (Wang et al., 2018), prioritizing cancer genes (Dimitrakopoulos et al., 2018), finding cancer drug targets (Valle et al., 2018), or discriminating cancer subtypes (Ramazzotti et al., 2018).

Cancer subtyping is one of the most important issues in cancer research. In precision medicine, patients need to be accurately diagnosed and treated based on their cancer subtypes due to the difference in therapeutic strategy and drug response. In recent years, many computational methods that integrate multi-omics data for cancer subtyping have been proposed. These approaches can be mainly divided into two categories based on data modeling strategies (Bersanelli et al., 2016), including graph-based approaches (Hofree et al., 2013; Wang et al., 2016; Guo et al., 2018) and statistics-based approaches (Shen et al., 2009; Yuan et al., 2011; Kim et al., 2017). However, even using the same dataset, different methods often generate inconsistent subtyping results. To our best knowledge, there is a lack of tools or platforms to compare these methods partly because there are no gold standards for evaluating their results. CancerSubtypes (Xu et al., 2017) is an R package that can be used to obtain cancer subtyping results by several existing methods. But it only implements basic evaluation on methods instead of their comparisons and also requires users to specify the exact number of subtypes to run each method, which is not easily determined in advance.

Since comprehensive evaluation and comparison between different methods serves as a useful tool or guideline for users to select an optimal method for their own purpose (Saelens et al., 2019), we develop CEPICS, a Comparison and Evaluation Platform for Integration methods in Cancer Subtyping. Using multi-omics data as input, CEPICS first evaluates subtyping results generated by up to five state-of-the-art data integration methods based on both clustering performance metrics and survival analysis. Then, CEPICS compares subtyping results across different methods and different numbers of subtypes. Finally, using all these results, CEPICS calculates a probability of belonging to the same subtype for each patient pair. Most importantly, CEPICS allows users to upload results of their own methods and compare with built-in methods. In the future, CEPICS will incorporate more published multi-omics integration methods by constantly updating. CEPICS is implemented as an R package and is freely available at https://github.com/GaoLabXDU/CEPICS. For convenience, we also construct a docker image of CEPICS. Please see **Supplementary Materials** for details.

## Results

Here, we introduce the framework of CEPICS and present its three different application scenarios based on genomics, transcriptomics, and epigenetics data.

### CEPICS Framework

**Figure 1:**
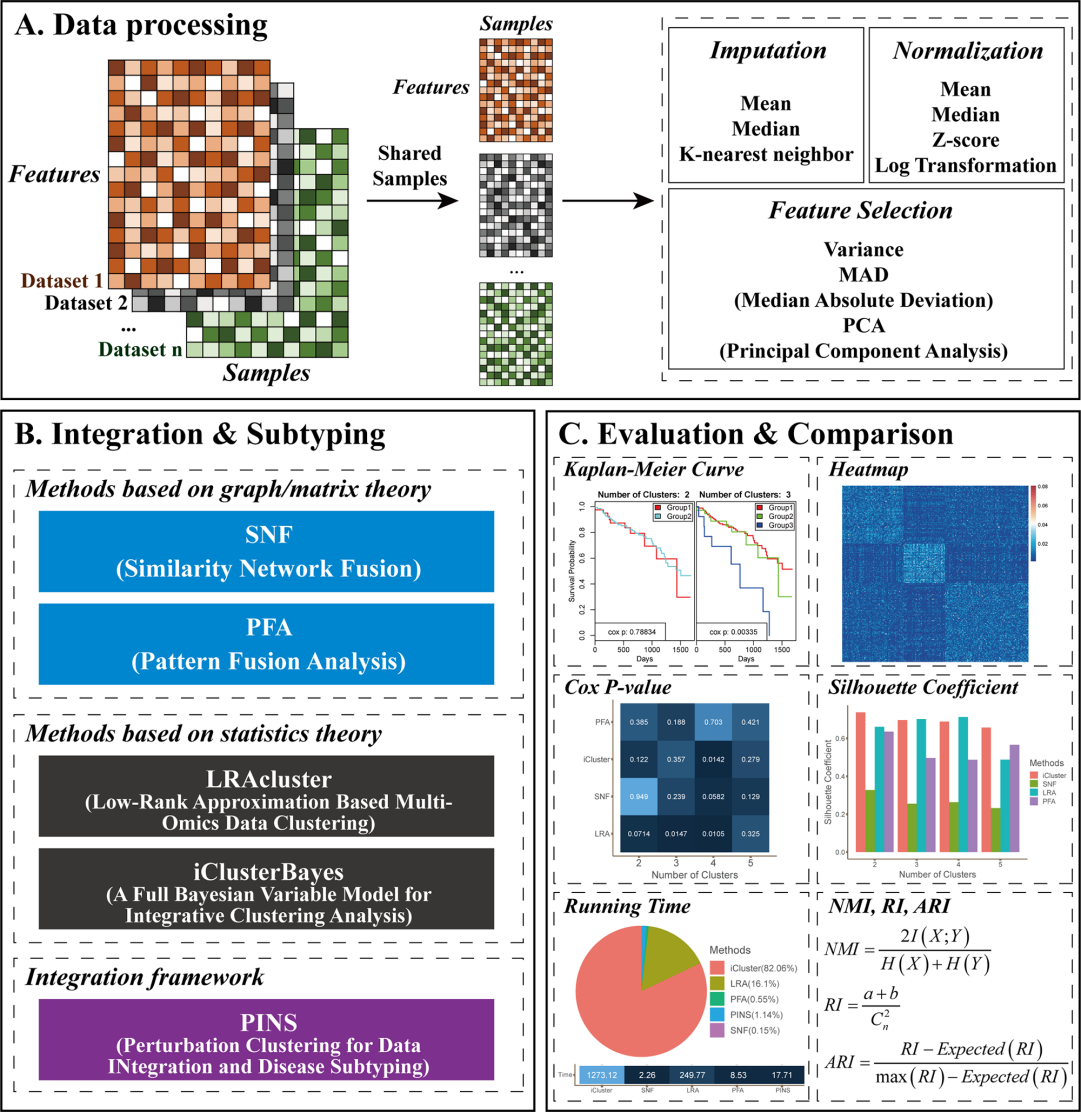
CEPICS framework. **(A)** Data processing step. Users can choose different strategies to impute missing values, select features, and normalize the data. **(B)** Integration and subtyping step. CEPICS utilizes five existing methods to obtain subtyping results. Users can select several or all of the methods. **(C)** Evaluation and comparison step. CEPICS evaluates results of each method using both clinical survival analysis and clustering performance metrics, and makes comparisons among the results vertically and horizontally.

Given two or more different types of omics data, CEPICS performs three steps to evaluate and compare subtyping results generated by up to five built-in integration methods. **Figure 1** shows an overview of the CEPICS framework. As the first step (**Figure 1A**), CEPICS requires users to upload a combination of at least two types of omics datasets (e.g., gene expression, DNA methylation, and copy number variants) as inputs. CEPICS first selects samples from different datasets that belong to the same patients. Then, users can choose one of three different ways [median/mean of each feature or *k*-nearest neighbor imputation (Troyanskaya et al., 2001)] to impute missing values. Four different strategies (e.g., z-score and log-transformation) are available to normalize the data to eliminate the differences in scales of different datasets. Moreover, users can choose variance, median absolute deviation (MAD), or principal component analysis (PCA) to select features for subtyping. Data imputation, normalization, and feature selection in this step are not necessary but recommended for better performance.

In the second step (**Figure 1B**), CEPICS provides users with five built-in methods to obtain cancer subtyping results. SNF (Wang et al., 2014) and PFA (Shi et al., 2017) are two representative methods based on graph/matrix theory. LRAcluster (Wu et al., 2015) and iClusterBayes (Mo et al., 2017) are two representative statistics-based methods. PINS (Nguyen et al., 2017; Nguyen et al., 2018) is an integration framework for disease subtyping using perturbation clustering. Due to the unknown true number (i.e., *k*) of cancer subtypes, CEPICS requests users to specify a maximum possible number of subtypes (i.e., *k*-max) so that CEPICS generates subtyping results from two to *k*-max respectively. In order to evaluate and compare the five built-in methods, we make some improvements on their method implementation; please see *Methods* for details.

Finally, for evaluation and comparison (**Figure 1C**), both survival analysis and clustering performance metrics are applied to each method, including Kaplan-Meier survival curves and p-value of the Cox proportional hazards model, similarity heatmap, silhouette coefficient, normalized mutual information (NMI), adjusted rand index (ARI), and time consumption. Moreover, an empirical pre-determination of subtypes for samples by experts or clinicians based on clinical phenotypes or images can be inputted to CEPICS and used as the gold standard.

CEPICS not only makes vertical comparisons on results generated by specifying different numbers of subtypes (i.e., *k*) in each method but also makes horizontal comparisons across different methods. Most importantly, as a scalable platform, CEPICS allows users to upload their own subtyping results so that it is very convenient to evaluate and compare their own methods to the state-of-the-art ones. For providing a high-confidence subtyping result, CEPICS employs a vote-based strategy to conduct a sample consensus clustering. The result represents a robust prediction for sample pairwise similarities because it considers all choice of *k* and all user-selected built-in methods.

When the evaluation and comparison completed, CEPICS generates a report which consists of two parts. The first part shows the comparison of subtyping results across different methods and different numbers of subtypes, including time consumption, Cox p-value, NMI, ARI, and silhouette coefficient. The overall sample similarity heatmap is also shown in this part. The second part shows the performance of each method, including summary information of the main metrics (when the true labels are available), KM survival curves, and patient similarity heatmaps for different numbers of subtypes in each method. CEPICS generates two versions of reports depending on the availability of true labels of patients.

### Case Studies

CEPICS is an easily-used R package. Here we present its three application scenarios using four different omics datasets of Colon Adenocarcinoma (COAD) from The Cancer Genome Atlas (TCGA), including mRNA expression, miRNA expression, DNA methylation, and copy number variation data.

For all application scenarios, CEPICS selected samples belonging to the same patient cohort for four omics datasets. Then CEPICS filtered the features that had more than 20% missing values across all patients and filtered the patients that had more than 20% missing values across all features for each dataset. *K*-nearest neighbor method was used to impute missing values and z-score was used to normalize each dataset. Top 10% features based on the MAD strategy were selected for subtyping. We set five as the maximum possible number of subtypes (i.e., *k*-max = 5) and chose all five built-in integration methods and all performance metrics for evaluation and comparison. CEPICS main results are elaborated below and complete reports are provided in **Supplementary Materials**.

#### Scenario 1: Comparisons Across Different *K* and Different Methods

Given multi-omics datasets without any subtype information, CEPICS uses each user-selected method to generate subtyping results based on *k* = 2 to *k*-max. Then, CEPICS evaluates and compares these results using both survival analysis and clustering performance metrics.

**Figure 2:**
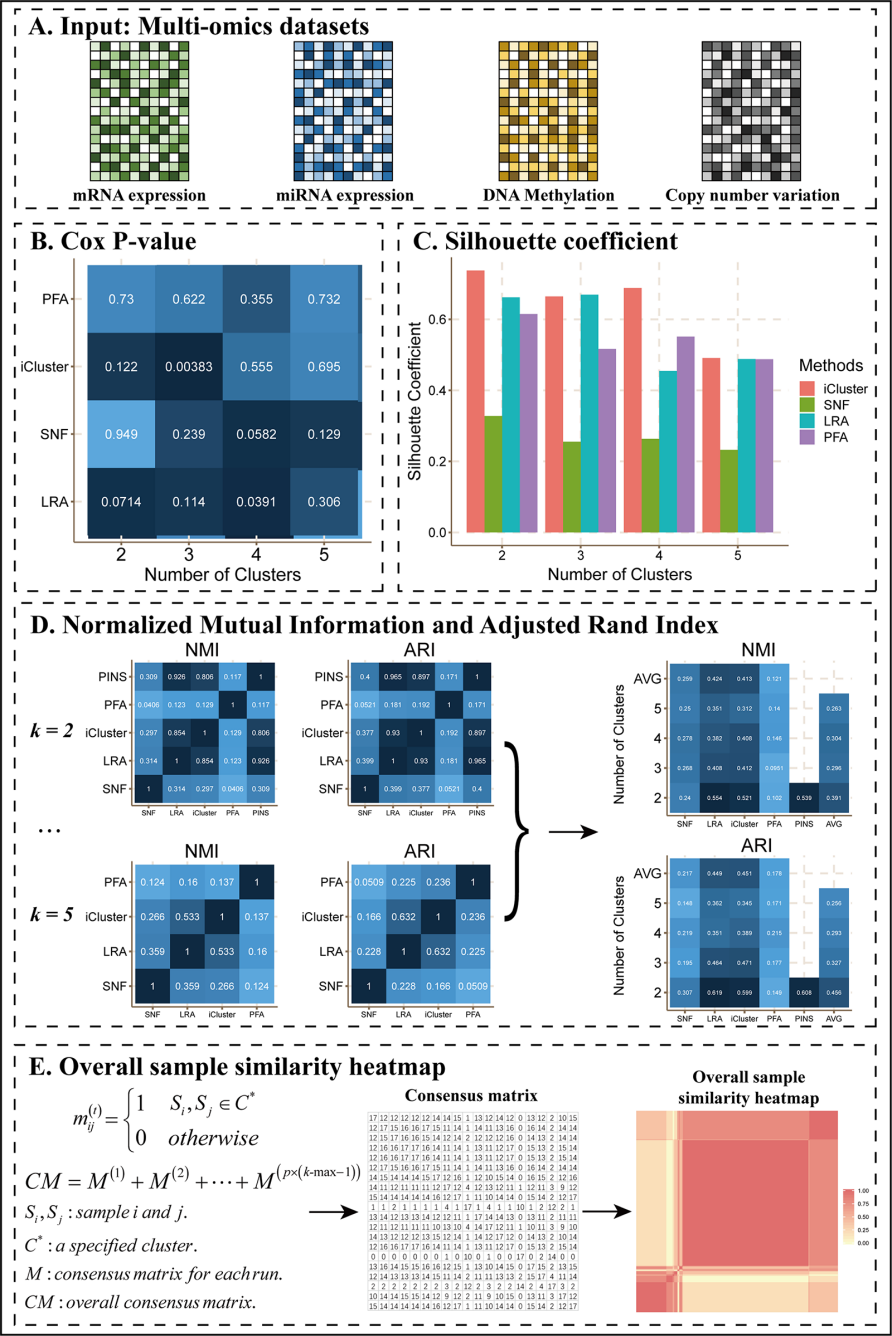
The main results of Scenario 1. **(A)** Data Input. Four datasets including mRNA expression, miRNA expression, DNA methylation, and CNV were uploaded to CEPICS. **(B)** Cox p-value heatmap showed the comparison based on clinical survival analysis across different methods and different numbers of clusters (subtypes) with p-values of the Cox proportional hazards model. The shade of color was inversely proportional to the p-value. **(C)** Silhouette coefficient comparison. **(D)** Comparisons of NMI and ARI. The shade of color in the NMI and ARI heatmaps was proportional to the consistency between results by different methods. Silhouette coefficient, NMI, and ARI showed the comparison based on clustering performance. **(E)** The process of generating the overall sample similarity heatmap.

As an example of this scenario (**Figure 2**), we applied CEPICS to four omics datasets of COAD (**Figure 2A**) and found that SNF, LRAcluster, and iClusterBayers had most significant Cox p-values at *k* = 4, 4, and 3, respectively (**Figure 2B**). However, there were no significant Cox p-values for the classification by PFA. Based on the silhouette coefficient, all methods achieved the best performance at *k* = 2 except LRAcluster which achieved the best performance at *k* = 3 (**Figure 2C**). Such inconsistency of optimal *k* suggested that multiple performance metrics should be considered simultaneously to avoid *k*-selection bias. To further examine the consistency of results between different methods and *k*, CEPICS calculated NMI and ARI between the results of every two methods for each *k*, and then calculated the average NMI and ARI for each method at each *k*. High NMI and ARI indicated that the result of this method had high agreements with others. We found that SNF and PFA had the highest NMI values at *k* = 4 while LRAcluster and iClusterBayes achieved that at *k* = 2. Meanwhile, all methods had the highest ARI values at *k* = 2 except PFA which achieved highest ARI at *k* = 4 (**Figure 2D**). Such inconsistency of subtyping results between different *k* and methods were also observed in other datasets (**Supplementary Files**). Motivated by this observation, we incorporated a consensus clustering step into CEPICS to help users obtain a robust subtyping result (please see *Methods* for details). Based on a voting strategy, CEPICS calculated a consensus matrix considering results of all methods and all choices of *k*, and then generated a consensus similarity heatmap by hierarchical clustering (**Figure 2E**).

#### Scenario 2: Comparisons Based on the Pre-Defined Subtyping Results

Given empirical pre-determination of subtypes for samples by experts, users may want to evaluate and compare the performance of integration methods based on pre-determined subtypes as true labels. For this purpose, CEPICS requires these true labels together with multi-omics data as inputs and calculates the NMI and ARI between the results by each method and the true labels.

A previous study (Sanchez-Vega et al., 2018) showed four subtypes of TCGA COAD patients defined by different TCGA analysis working groups. Here, we considered them as true labels and uploaded them with COAD datasets to CEPICS.

**Figure 3:**
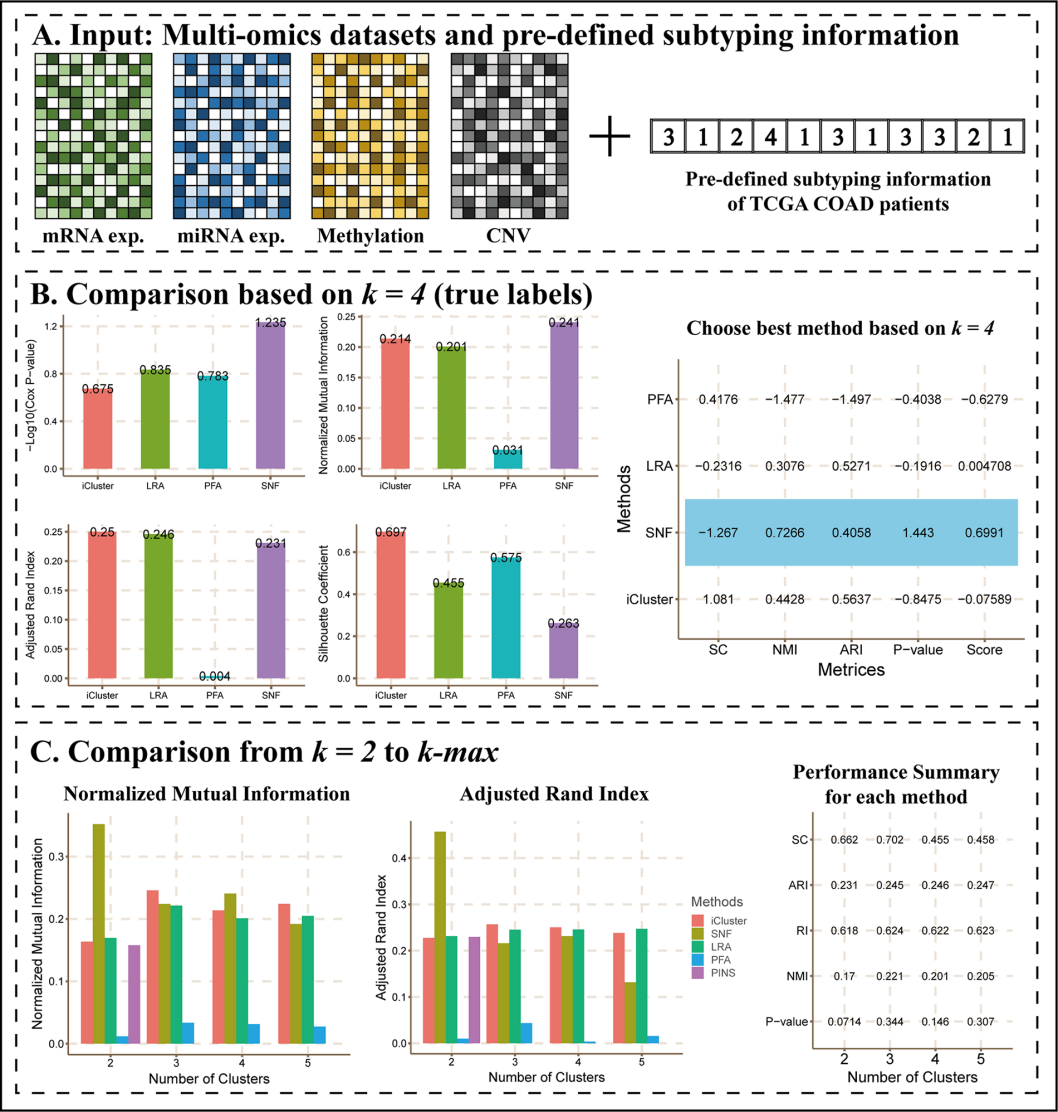
The main results of Scenario 2. **(A)** Data Input. A pre-defined subtyping result with four datasets was uploaded to CEPICS. CEPICS took the pre-defined subtyping result as the gold standard to evaluate each method, and then made comparisons. **(B)** Comparisons based on *k* = 4. CEPICS suggested the best method according to Cox p-value, NMI, ARI and silhouette coefficient (SC) comparisons based on *k* = 4. The method which had the highest score (SNF for this case) was considered to be the best method based on the current *k*, and was highlighted by blue in the table. **(C)** Comparisons from *k* = 2 to *k*-max. After comparing, CEPICS summarised the performance of the main metrics for each method for all choices of *k* in the second part of the report.

**Figure 3** shows the main results of Scenario 2. CEPICS first made comparisons of Cox p-values, NMI, ARI, and silhouette coefficient based on *k* = 4 which was the number of pre-determined subtypes (**Figure 3B**). We found that SNF had the highest –log_10_ (Cox p-value) and NMI, while iClusterBayes had the highest ARI and silhouette coefficient. In this situation, considering both clustering and clinical metrics, CEPICS set different weights for four metrics and scored each method to suggest the best one (please see *Methods* for details). Then, CEPICS made comparisons across different methods from *k* = 2 to *k*-max (**Figure 3C**). For detailed performance comparisons, please see CEPICS-generated complete reports.

#### Scenario 3: Comparing the User’s Own Method With Built-In Ones

Researchers have been developing new cancer subtyping methods. Using CEPICS, it is very convenient to evaluate user’s own subtyping method and make comparisons to other state-of-the-art methods built in CEPICS. For this purpose, CEPICS requires the results of user’s own method together with multi-omics data as inputs. In order to generate heatmaps and calculate silhouette coefficients, CEPICS needs an integrated data matrix which can be a patient-patient similarity matrix, a patient-patient distance matrix, or a patient-feature matrix after integration. If these matrices are not available, CEPICS can perform analysis based on subtyping results only as Scenario 2. Furthermore, we can also upload pre-determined subtype information as true labels for evaluations and comparisons.

**Figure 4:**
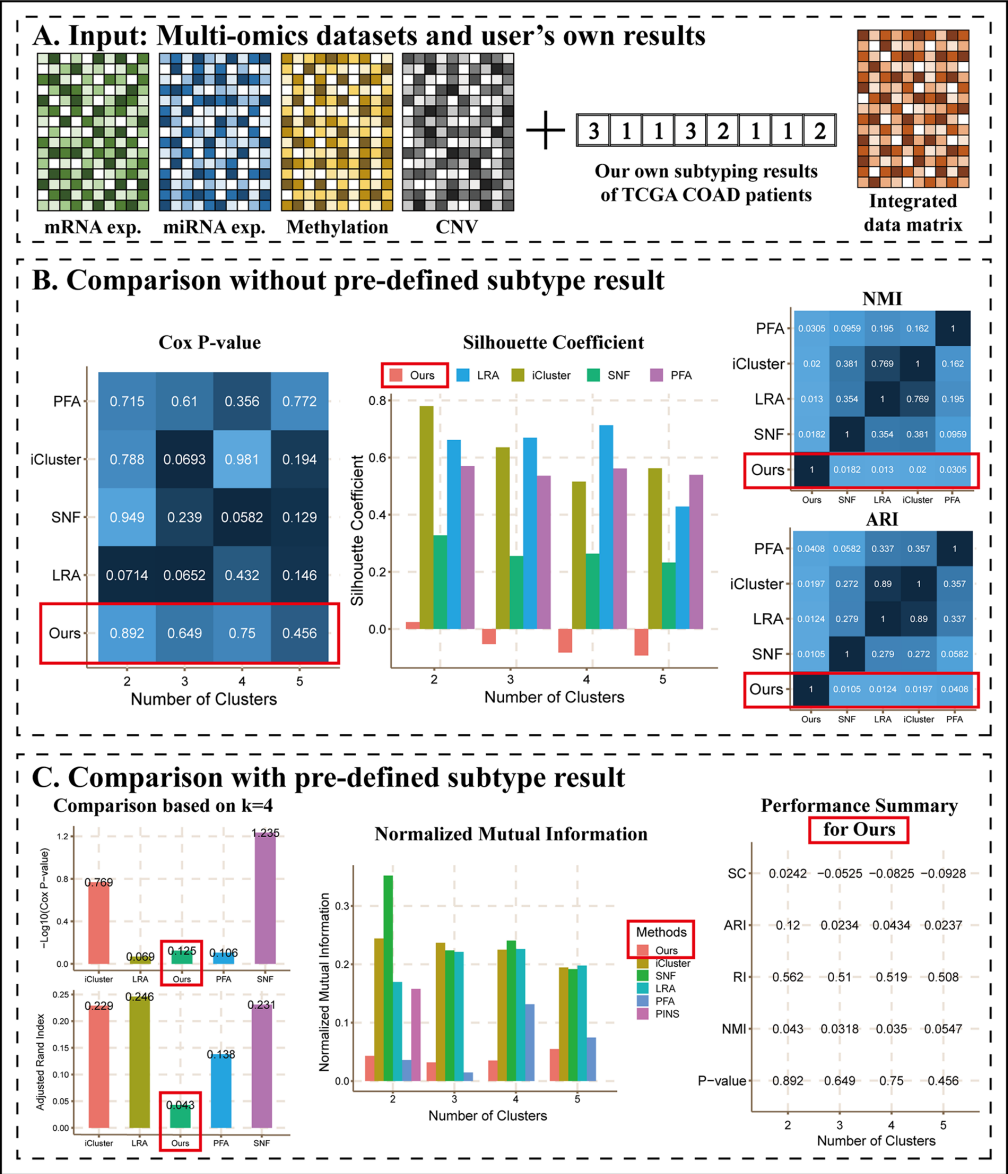
The main results of Scenario 3. **(A)** Data Input. Subtyping results of our own method and an integrated data matrix were uploaded with four datasets to CEPICS. CEPICS compared our results with other built-in methods. **(B)** Comparisons without pre-defined result. **(C)** Comparisons with the pre-defined result. We uploaded the pre-defined result in Scenario 2 to make comparisons based on it. The performances of ‘Ours’ were highlighted by red boxes.

As an example of this scenario (**Figure 4**), we applied *k*-means to perform clustering on COAD mRNA expression dataset using *k* = 2 to 5, respectively, and we considered them as results of the user’s own method, denoted as “Ours.” Then, we calculated a patient-patient similarity matrix as the integrated data matrix. We did not upload true labels as described in Scenario 1 and ran CEPICS for the first time, and then we uploaded them and reran. Thus, we generated two reports for Scenario 3 (**Supplementary Files**).

## Discussion

We developed CEPICS, a scalable and easily-used R package, to provide a comparison and evaluation platform for multi-omics data integration methods in cancer subtyping. Using CEPICS, researchers can easily obtain subtyping results by state-of-the-art methods and make extensive evaluation and comparisons among them. Based on the observation that subtyping results have high dependencies on the number of subtypes and low consistency between different methods, CEPICS generates a robust result of sample similarity prediction and shows the overall sample similarity heatmap.

Compared with CancerSubtypes (Xu et al., 2017), CEPICS focuses on performing a more comprehensive evaluation and comparison of subtyping results. Besides choosing representative methods based on two data modeling approaches, there are also three main differences described below:

1. *k*-max instead of *k*. CancerSubtypes requires users to specify the exact number of subtypes to run each method, which is not easily determined in advance. Due to the unknown true number (i.e., *k*) of cancer subtypes, CEPICS requests users to define a maximum possible number of subtypes (i.e., *k*-max) instead of *k* in order to perform a vertical comparison between different *k* ranging from two to *k*-max, which comprise a large amount of work.
2. Overall sample analysis. CEPICS employs a vote-based strategy to give a sample consensus clustering result, which represents a robust prediction for sample pairwise similarities because it considers all choices of k and all user-selected methods.
3. Comparison with existing subtyping results. CEPICS allows users to upload their own subtyping results so that it is very convenient for researchers to evaluate and compare their own methods to the state-of-the-art ones. Moreover, an empirical pre-determination of subtypes can also be inputted to CEPICS, which can be used as the gold standard to evaluate and compare results generated by different methods.

Using CEPICS to make comparisons between different methods, we found that the two statistics-based methods, iClusterBayes and LRAcluster, were relatively inefficient. For example, the proportion of time consumption for iClusterBayes shown in the pie charts of reports were all over 80% (**Supplementary Files**). The most important reason is that there are many parameters in statistics-based methods and the training process may cost much time. Parallel computation makes these methods more efficient. In summary, CEPICS represents a useful integrated tool for cancer subtyping using multi-omics data and will be updated constantly as data integration methods accumulate.

## Methods

In this section, we describe the technical details of CEPICS. First, we introduce the five built-in methods and their improvements by us, especially for LRAcluster. Then, performance evaluation metrics are provided from different views. Upon these evaluations, we describe an approach for constructing an overall sample similarity heatmap to illustrate a consensus subtyping result of samples. Finally, we illustrate a strategy that determines the best method based on true labels.

### Built-In Integration Methods and Improvements

We incorporate five multi-omics integration methods for subtyping into CEPICS, which can be classified into three categories. The first category includes two network/graph model-based methods. Similarity Network Fusion (SNF) constructs a sample-sample similarity network for each data type and then integrate them based on message-passing theory. Spectral clustering is performed on the integrated similarity network to discriminate disease subtypes. Pattern Fusion Analysis (PFA) aligns local sample-pattern from each data type into a global sample-pattern based on an adaptive optimization strategy and then uses k-means clustering to identify subtypes. There are also two statistics-based methods in the second category. Low-rank Approximation Based Multi-omics Data Clustering (LRAcluster) uses a low-rank approximation-based probabilistic model to integrate different omics data and then performs k-means clustering to identify disease subtypes. iClusterBayes uses the Bayesian latent variable regression model to integrate different omics data and also performs k-means clustering to discriminate subtypes. Perturbation Clustering for Data Integration and Disease Subtyping (PINS) is an integration framework as the third category. It considers the resilience of patient connectivity and cluster ensembles to integrate different omics data, and then uses hierarchical clustering, PAM or Dynamic Tree Cut to identify subtypes. Since both PFA and LRAcluster packages only output an integrated sample-feature matrix after data integration rather than clustering (subtyping) results, CEPICS uses *k*-means with 300 iterations to obtain stable subtyping results.

In order to evaluate and compare the five built-in methods, we format inputs and outputs of all methods consistently and also make some improvements on them.

When using the LRAcluster R package for dimension reduction, users must manually determine the dimension of the reduced subspace used for clustering according to an explained variance (EV) plot. For convenience, CEPICS implements an automatic procedure to perform dimension reduction.

**Figure 5:**
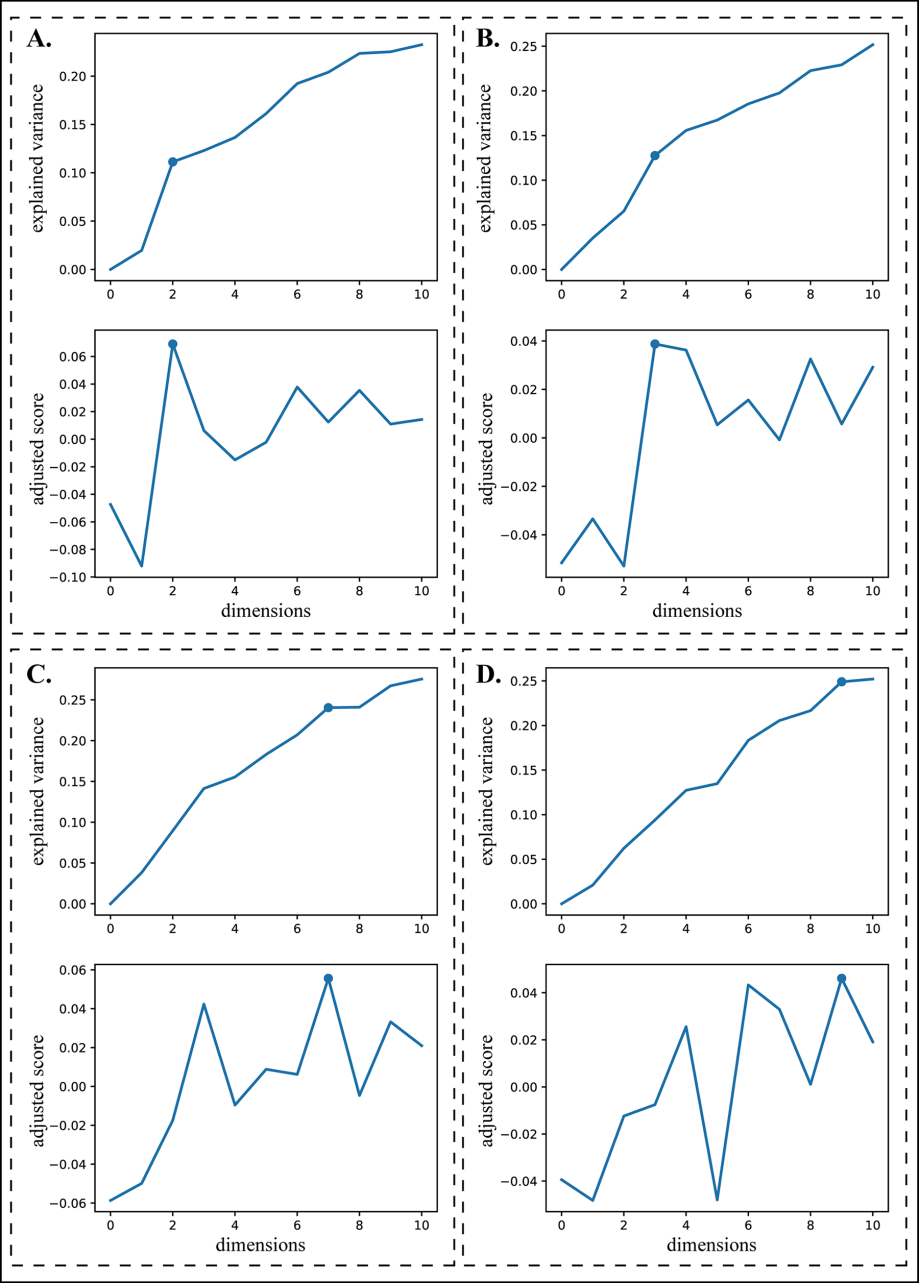
Turning points selection. The upper plot of each panel is an explained variance plot and the lower one is a corresponding adjusted turning score plot. **(A)** and **(B)** illustrate two situations where the turning points can be easily chosen manually. **(C)** and **(D)** illustrate two situations where the turning points are difficult to choose manually.

An EV plot (**Figure 5**) describes the explained variances for reduced dimensions from 1 to *n*, a user-specified maximum dimension (default as 10). Typically, the reduced dimension can be determined based on a turning point on the EV curve according to the criterion that the increase of model fitness is much slower after it (Wu et al., 2015). Suppose that there are *n* points on the curve. For point *i*, we define a relative difference in EV values between any other point *j* and *i* as

$$h(i,j)=\{\begin{array}{cc} \frac{\mathrm{ev}(j)-\mathrm{ev}(i)}{{\left|x(j)-x(i)\right|}^{\delta }} & x(i)\neq x(j)\\ 0 & x(i)=x(j) \end{array}$$

where *x*(·) is the x-coordinate (i.e., the reduced dimension) of point *i* or *j*, and *ev*(·) is the EV value of that point. *δ* is a parameter which controls the impact of the difference in x-coordinates between points *i* and *j* on their relative difference in EV values, and *δ* ≥ 0. When *δ* = 0, *h*(*i,j*) represents their difference in EV values. When *δ* = 1, *h*(*i,j*) indicates the slope of the curve between points *i* and *j* when *x*(*j*) > *x*(*i*), and the negative slope when *x*(*j*) < *x*(*i*). When *δ* > 1, the larger difference in x-coordinates between the two points, the smaller their relative difference in EV values. To consider the global information, we set *δ* = 2.2. Then, we define a turning score as

$$\mathrm{turning}\_\mathrm{score}\;(i)\text{ = }\sum_{k=1}^{i-1}h(k,i)-\sum_{k=i+1}^{n}h(i,k)=\sum_{k=1}^{n}h(k,i)$$

To avoid the bias of the turning score on terminal points of the EV curve, we introduce a penalty to each point *i*, defined as $\mathrm{penalty}(i)=\exp\left(-\frac{{\left(x(i)-\mu \right)}^{2}}{5v}\right)$, where *µ,v* are the mean and the variance of x-coordinates, respectively. Then, an adjusted turning score is defined as

adjusted_turning_score(i)=score(i)×penalty(i)

The x-coordinate of the turning point *i* ^*^ with the highest adjusted turning score is chosen as the reduced dimension. **Figure 5** shows four cases of selecting the turning points, demonstrating that this procedure can automatically choose the turning points appropriately even for the curves in which they are difficult to determine manually (**Figures 5C, D**).

The process of choosing the reduced dimension can be parallel computed. We bring the parallel computation to LRAcluster which can save much time. iClusterBayes can be parallel computed only on Linux system or macOS because of the limitation of parallel function the authors used. We employ a different parallel function that is also supported on Windows system and make it possible to run iClusterBayes in parallel without operating system limitation.

Since only the MATLAB source code is available for the PFA algorithm, CEPICS uses R.matlab R package to initialize a MATLAB server to run PFA. The principle of the PFA method has no limitation on the number of datasets. However, the code provided by the author requires three datasets as input. We improve the code in order to integrate multi-omics data without the limitation of the number of datasets by modifying the structure of the code to fit different numbers of datasets and calling the PFA core code as the author did.

### Performance Evaluation Metrics

For comprehensive evaluation and comparison, both survival analysis and clustering performance metrics are applied to each method.

1. **Kaplan-Meier survival curves and p-value of the Cox proportional hazards model** evaluate the significance of difference among different subtypes identified by a method. Generally, *p–value* < 0.05 suggests that subtyping results are reasonable.
2. **Similarity heatmap** shows similarity patterns of patient samples.
3. **Silhouette coefficient** measures the similarity between one sample and its classified subtype compared to other subtypes to determine how appropriately the samples have been clustered. For a sample *i*, the silhouette coefficient can be calculated as $s(i)\text{ = }\frac{b(i)-a(i)}{\max\{a(i),b(i)\}}$, where *a*(*i*) is the average distance between *i* and other samples in its own cluster, *b*(*i*) is the minimum distance between *i* and other samples which are not in its own cluster. Then, the silhouette coefficient of the result can be defined as $\mathrm{SC}=\frac{1}{n}\sum_{i=1}^{n}s(i)$, where *n* is the total number of samples. *SC* has a range [-1, 1] and higher values represent better performance.
4. **Normalized mutual information (NMI)** measures the concordance of two subtype results. It can be calculated as $U(X,Y)=\frac{2I(X;Y)}{H(X)+H(Y)}$, where H(·) represents the entropy of a cluster, and I(X;Y) represents the mutual information of X and Y. U(X,Y) has a range [0, 1] and higher values represent better performance.
5. **Adjusted rand index (ARI)** measures the agreement between two subtype results. Suppose that we have two partitions, *U*={*u*_1_,*u*_1_,…,*u_r_*} and *V*={*v*_1_,*v*_2_,…,*v_s_*}, of *N* samples. We take U as true labels and V as a clustering result. A matrix can be constructed with each entry *n_ij_* is the number of samples that are both in clusters *u_i_* and *v_j_*, *i*=1,2,…,*r*, *j* = 1,2,…,*s*. Define $n_{i\cdot }=\sum_{j=1}^{s}n_{\mathrm{ij}}$ and $n_{j\cdot }=\sum_{i=1}^{r}n_{\mathrm{ij}}$ denote the sum of row *i* and column *j*, respectively, and $V=\left(\begin{array}{c} N\\ 2 \end{array}\right)=\frac{N(N-1)}{2}$. Then, the ARI of the two partitions *U* and *V* is defined as (Hubert and Arabie, 1985; Dudoit and Fridlyand, 2002; Wu et al., 2005):
  $$\mathrm{ARI}=\frac{\sum_{i=1}^{r}\sum_{i=1}^{s}\left(\begin{array}{c} n_{\mathrm{ij}}\\ 2 \end{array}\right)-\frac{1}{V}\sum_{i=1}^{r}\left(\begin{array}{c} n_{i\cdot }\\ 2 \end{array}\right)\sum_{i=1}^{s}\left(\begin{array}{c} n_{\cdot j}\\ 2 \end{array}\right)}{\frac{1}{2}\left[\sum_{i=1}^{r}\left(\begin{array}{c} n_{i\cdot }\\ 2 \end{array}\right)+\sum_{i=1}^{s}\left(\begin{array}{c} n_{\cdot j}\\ 2 \end{array}\right)\right]-\frac{1}{V}\sum_{i=1}^{r}\left(\begin{array}{c} n_{i\cdot }\\ 2 \end{array}\right)\sum_{i=1}^{s}\left(\begin{array}{c} n_{\cdot j}\\ 2 \end{array}\right)}.$$
  The *ARI* has a range [-1, 1] and higher values represent better performance. *ARI*=1 represents that the clustering result is perfectly matched with the true labels.
6. **Time consumption** illustrates the running time of each method.

### Overall Sample Similarity Heatmap

**Figure 2E** shows the process of generating an overall sample similarity heatmap. If *p* methods are selected by a user, CEPICS will perform *p*×(*k*-max–1) runs in total. CEPICS calculates a consensus matrix $M_{n\times n}^{\left(t\right)}$ for each run where *n* is the number of samples, and $m_{ij}^{\left(t\right)}=1$ only when the sample *i* and *j* are clustered in the same cluster, otherwise, $m_{ij}^{\left(t\right)}=0$. After all runs, CEPICS calculates an overall consensus matrix $\mathrm{CM}=\sum_{t=1}^{p\times (k\text{-}\max-1)}M^{\left(t\right)}$, and *cm_ij_*∈[0,*p*×(*k*-max–1)]. Applying hierarchical clustering to *CM*, CEPICS generates a clustering result which represents a robust prediction for sample pairwise similarities because it considers all choices of *k* and all user-selected methods.

### Finding the Best Method Based on True Labels

When a user uploads a pre-determined subtyping result, CEPICS considers it as the gold standard to make comparisons and suggests the best method according to the combination of the Cox p-value, NMI, ARI and silhouette coefficient. For a performance metric *m*, CEPICS calculates *z*-score of the result *x*(*i*) of each method *i* as $z{\left(i\right)}_{m}=\frac{x(i)-\mu }{\sigma }$, where *µ* and *σ* are the means and standard deviations of all results of the metric *m*, respectively. For the Cox p-value, CEPICS transforms $x{\left(i\right)}^{*}=-\log_{10}x(i)$ and then calculates *z*-scores. Considering that both clinical survival analysis and clustering performance metrics should have the same weight, a performance score for each method *i* is defined as

$$\mathrm{performance\_score}(i)=\alpha z{\left(i\right)}_{\mathrm{Cox}\;p-\mathrm{value}}+\frac{\beta }{3}(z{\left(i\right)}_{\mathrm{NMI}}+z{\left(i\right)}_{\mathrm{ARI}}+z{\left(i\right)}_{\mathrm{silhouette}\;\mathrm{coefficient}})$$

where α=β=0.5. CEPICS suggests the best method with the highest performance score.

## Data Availability Statement

The source code and binaries of CEPICS, and the demo datasets are available at https://github.com/GaoLabXDU/CEPICS; Project name: CEPICS; Operating system(s): Windows, Linux, and macOS; Programming language: R; License: General Public License (GNU GPLv3); Any restrictions to use by nonacademics: None. The datasets analyzed for this study are available at the Genomic Data Commons Data Portal: https://portal.gdc.cancer.gov/

## Author Contributions

RD and LG conceived the study. HX and KS developed the program and wrote the codes. RD, HW, YD, CZ, and SJ tested the R package. RD wrote the manuscript. YH, LG, and RD revised the manuscript. All authors read and approved the manuscript.

## Funding

This work was supported by the National Key R&D Program of China No. 2018YFC0910400 to LG, the National NSFC (Grant No. 61532014 and No. 61432010 to LG, and No. 61702397 to SJ), and the Shanghai Municipal Science and Technology Major Project (No.2018SHZDZX01), LCNBI, and ZJLab.

## Conflict of Interest

The authors declare that the research was conducted in the absence of any commercial or financial relationships that could be construed as a potential conflict of interest.

## Supplementary Material

The Supplementary Material for this article can be found online at: https://www.frontiersin.org/articles/10.3389/fgene.2019.00966/full#supplementary-material and https://github.com/GaoLabXDU/CEPICS.
