# Supplementary material for: CEPICS: A Comparison and Evaluation Platform for Integration Methods in Cancer Subtyping
Source: Front Genet. 2019 Oct 8;10:966. doi: 10.3389/fgene.2019.00966 (PMC6792302; doi:10.3389/fgene.2019.00966)
Supplement: Supplementary file 1 [file DataSheet_1.zip › CEPICS Supplementary materials/BRCA-1.html]

Evaluation and Comparison Report


# Evaluation and Comparison Report

#### 2019-08-31 11:59:34

# Time Consumption

# Cox P-value

# Normalized Mutual Information and Adjusted Rand Index

## Comparison Based on 2 Clusters

## Comparison Based on 3 Clusters

## Comparison Based on 4 Clusters

## Comparison Based on 5 Clusters

# Silhouette Coefficient

# Samples Similarity HeatMap

========================================================================================

========================================================================================

# Performance of Each Method

## LRAcluster

### Kaplan-Meier Survival Curves

### HeatMaps

#### Number of Clusters: 2

#### Number of Clusters: 3

#### Number of Clusters: 4

#### Number of Clusters: 5

## SNF

### Kaplan-Meier Survival Curves

### HeatMaps

#### Number of Clusters: 2

#### Number of Clusters: 3

#### Number of Clusters: 4

#### Number of Clusters: 5

## iClusterBayes

### Kaplan-Meier Survival Curves

### HeatMaps

#### Number of Clusters: 2

#### Number of Clusters: 3

#### Number of Clusters: 4

#### Number of Clusters: 5

## PFA

### Kaplan-Meier Survival Curves

### HeatMaps

#### Number of Clusters: 2

#### Number of Clusters: 3

#### Number of Clusters: 4

#### Number of Clusters: 5

## PINS

### Kaplan-Meier Survival Curves
