# Supplementary material for: CEPICS: A Comparison and Evaluation Platform for Integration Methods in Cancer Subtyping
Source: Front Genet. 2019 Oct 8;10:966. doi: 10.3389/fgene.2019.00966 (PMC6792302; doi:10.3389/fgene.2019.00966)
Supplement: Supplementary file 1 [file DataSheet_1.zip › CEPICS Supplementary materials/CEPICS Supplementary materials.doc]

***Supplementary Materials***

**CEPICS: a Comparison and Evaluation Platform for Integration Methods in Cancer Subtyping**

**1.**	**Guideline for CEPICS R package**	1

1.1	Install CEPICS	1

1.2	Data pre-processing	2

1.3	Feature selection	2

1.4	Integration, evaluation, and comparison	2

**2.**	**Guideline for docker version of CEPICS**	4

**3.**	**Index of Generated Files**	5

**Reference**	6

1. **Guideline for CEPICS R package**

CEPICS is an easily-used R package to provide a comparison and evaluation platform for multi-omics data integration methods for cancer subtyping.

1.1 **Install CEPICS**

You can download CEPICS from [https://github.com/GaoLabXDU/CEPICS](https://github.com/GaoLabXDU/CEPIM) and install the package from the local file and then library CEPICS.

Before installing CEPICS, some essential packages listed below should be installed, and you can source “init.R” ([https://github.com/GaoLabXDU/CEPICS/raw/master/documents/init.R](https://github.com/GaoLabXDU/CEPIM/raw/master/documents/init.R)) to install these packages except “ggthemr”.

- CRAN: cluster, devtools, DMwR, flexclust, ggplot2, pheatmap, PINSPlus, R.matlab, rmarkdown, SNFtool, survival.
- Bioconductor: iClusterPlus
- Others
  - LRAcluster (Download from <http://bioinfo.au.tsinghua.edu.cn/member/jgu/lracluster/> or <http://lifeome.net/software/lracluster/>)
  - ggthemr (install devtools package first.)

install.packages(“devtools”)

devtools::install_github('cttobin/ggthemr')

1.2 **Data pre-processing**
- **Load data.** Use **formatData()** to load your data. Two types of data are the minimum requirement. Each type of data should be formatted into a matrix with columns as samples (e.g, patients) and rows as features (e.g. genes). You can use the parameter “trans” to [transpose](javascript:;) [the](javascript:;) data [matrix](javascript:;).

>mRNA <- formatData("COAD_mRNA.csv", trans=FALSE, sep=',')

You can also load the COAD data in CEPICS.

>data("COAD_mRNA")

- **Filter features and samples.** Use **filterData()** to filter features or samples according to the ratio of missing value to one feature or sample. If you want to treat zeros as NA, set the "filter_zero_as_NA" parameter as TRUE.

> filteredmRNA <- filterData(COAD_mRNA, alpha1 = 0.8, filter_zero_as_NA= FALSE)

- **Get overlap samples.** Use **getOverlap()** to selects samples from different omics datasets that belong to the same patient cohort.

>data("COAD_miRNA")

>datalist <- getOverlap(list(COAD_mRNA, COAD_miRNA))

- **Data imputation.** CEPICS provides three methods to impute the missing data: impute with the mean value of each feature, impute with the median value of each feature, and *k*-nearest neighbor method. If you want to impute zeros and NA, set "trans_zero_to_NA" as TRUE.

> mRNA <- imputeData(COAD_mRNA, method = 'knn', k = 10, trans_zero_to_NA = FALSE)

- **Data normalization.** CEPICS provides four methods to normalize the data: minus the mean value of each object, minus the median value of each object, calculate z-score for each data, and log-transformation. The parameter "object" can be chosen as sample or feature.

> mRNA <- normData(mRNA, method = "z_score", object = "sample")

1.3 **Feature selection**

CEPICS provides three criteria to select features: based on variance, MAD, and PCA.

> mRNA <- selectFeature (mRNA, method = "MAD", select_number = 0.9)

1.4 **Integration, evaluation, and comparison**

There are five built-in methods in CEPICS: SNF, PFA, iClusterBayes, LRAcluster, and PINS, and you can select part or all of them to obtain cancer subtyping results. Each method has its own parameter settings, you can use default or your own settings. CEPICS should be specified *k*-max which means the possible maximum number of subtypes, then runs each method to identify two to *k*-max subtypes. **Please make sure that MATLAB has been installed on your computer if you want to run PFA.**

We notice that when we run PINS using PINSPlus R package (Nguyen et al., 2018), the integrated result matrix is not generated so that both heatmap and silhouette coefficient are not available for PINS. Moreover, PINS generates subtyping results automatically and cannot be specified the number of subtypes. Therefore, CEPICS will compare PINS to other methods only based on the k corresponding to its results.

CEPICS provides six metrics to evaluate and compare the results generated by different methods: Kaplan-Meier survival curves and Cox p-value, Similarity heatmap, Silhouette coefficient, NMI, ARI, and Time consumption.

In order to generate Kaplan-Meier survival curves and calculate Cox p-values, clinical survival data should be provided as input and formatted as a data matrix. The names of columns are “name” (e.g. TCGA patients barcode), “OS_Days” which means survival time, and “OS_Status” which means sample status. You can also provide age and gender information and name the corresponding columns as “Gender” and “Age”, respectively. If age and gender information is available, CEPICS will choose the patient group (based on cancer subtypes), age, and gender as covariates to fit a Cox model and calculate an adjusted Cox p-value for patient groups. Otherwise, CEPICS will consider the patient group factor only in the Cox model. You can load “KMSURDATA” to see the example. For convenience, CEPICS has already collected clinical data of five cancers (BRCA, COAD, KIRC, LUAD, and LUSC) from TCGA with all factors. There is no need to set “KMdata” parameter when research on these five cancers and CEPICS will match the TCGA sample barcodes (15 bits) of the input data with the barcodes of clinical data.

>clinicaldata<- formatData("clinical_data.csv", trans=FALSE, sep=',')

>CEPICS(… , KMdata = clinicaldata, …)

If you don’t have clinical survival data, you can evaluate and compare methods without Kaplan-Meier survival curves and Cox p-values.

>CEPICS(… , KMdata = FALSE, …)

Here, we list some examples according to different scenarios.

> data("COAD_cnv")

> data("COAD_Methy")

> data("COAD_miRNA")

> data("COAD_mRNA")

> datalist <- list(COAD_cnv, COAD_Methy, COAD_miRNA, COAD_mRNA)

- **Scenario 1**

>CEPICS(datalist, c("gaussian", "gaussian", "gaussian", "gaussian"), functionList = list('PINS', 'LRA', 'SNF', 'PFA', 'iCluster'), kMax = 5)

- **Scenario 2**

If you have the empirical pre-determination of subtypes for samples, you can upload them, and CEPICS will take them as the true labels (gold standard) to evaluate and compare the results generated by different methods and different numbers of subtypes. Here, we take the subtypes of COAD defined by TCGA previously (Sanchez-Vega et al., 2018) as true labels.

>data("truelabel")

>CEPICS(datalist, c("gaussian", "gaussian", "gaussian", "gaussian"), functionList = list('PINS', 'LRA', 'SNF', 'PFA', 'iCluster'), kMax = 5, trueLabel = truelabel)

- **Scenario 3**

If you have the results generated by your own method, you can upload them to CEPICS to compare with other methods. The results should be formatted as a list, including “data”, “name”, and “time”. “data” is also a list, including “cluster” and “data”. “Cluster” is a sample-size × (*k*-max-1) matrix, it shows the cluster assignments of each sample from *k*=2 to *k*-max. “Data” is a sample-sample similarity matrix or sample-feature matrix. “time” is the float number represents the running time of your own method. “name” is the name of your method. You can load “Res” to see the example. Here, we use *k*-means to cluster COAD mRNA expression data. We set *k*-max as 2 to 5 and run *k*-means method respectively. Then we take the results as the results of our own method. We calculate the patient-patient similarity matrix as the integrated data matrix.

>data("Res")

>CEPICS(datalist, c("gaussian", "gaussian", "gaussian", "gaussian"), functionList = list('PINS', 'LRA', 'SNF', 'PFA', 'iCluster'), kMax = 5, Res = Res)

We can also upload the subtype information in scenario 2 together to run CEPICS.

> CEPICS(datalist, c("gaussian", "gaussian", "gaussian", "gaussian"), functionList = list('PINS', 'LRA', 'SNF', 'PFA', 'iCluster'), kMax = 5, trueLabel = truelabel, Res = Res)

2. **Guideline for docker version of CEPICS**

If you have a docker environment on your computer, you can run CEPICS easier. We have constructed a docker image which includes R-studio, the essential R packages for CEPICS, and CEPICS R package. We have pushed this image to the docker hub. Users can pull and run this docker image as:

docker pull rduanxdu/cepics:latest	# pull CEPICS docker image

docker run -e PASSWORD=rstudio -p 8787:8787 rduanxdu/cepics	# run it

After successfully running the image, users can use a browser to open the R-studio server by open the link as “localhost:8787”. The username is “rstudio”, and the password is user-defined when running the image. Then users can library(CEPICS) to use CEPICS illustrated in section 1.

It is also worth noting that the PFA method needs a MATLAB environment to run. When we constructed the current docker version of CEPICS, we failed to install the MATLAB environment. Then, we tried to transform the PFA method from MATLAB code to R code, but we found that the PFA results derived from a left division operation on non-square matrices in R and MATLAB were inconsistent. Therefore, in the current docker version, the PFA method cannot be used while the other four methods are ready to run.

3. **Index of Generated Files**

CEPICS saves all the subtyping and comparison results in the folder named as “CEPICS_yyyymmddhhmmss” in the current working directory which can be found using getwd(). All the figures are saved in “fig” folder. Table S1 shows the index and description of the generated files.

Table S1 Index and description of the generated files

| **Filename** | **Scenarios** | | | | **Description** |
| --- | --- | --- | --- | --- | --- |
|  | **S1** | **S2** | **S3** | **S3*** |  |
| **CEPICS.html** | **√** | **√** | **√** | **√** | **The comparison and evaluation report.** |
| ARI_SNF.csv |  | **√** |  | **√** | The ARI value of SNF results comparing to the true labels from k=2 to *k*-max. |
| NMI_SNF.csv |  | **√** |  | **√** | The NMI value of SNF results comparing to the true labels from k=2 to *k*-max. |
| RI_SNF.csv |  | **√** |  | **√** | The RI value of SNF results comparing to the true labels from k=2 to *k*-max. |
| sampleClu_2_x.csv | **√** | **√** | **√** | **√** | Consensus matrix which shows how many methods cluster each pair patients into the same cluster when k=2. This file will be generated in the situation of k=2 to *k*-max. |
| SI_SNF.csv |  | **√** |  | **√** | The silhouette coefficient value of SNF results from k=2 to *k*-max. |
| SNF_cluster.csv | **√** | **√** | **√** | **√** | The subtyping results of SNF from k=2 to *k*-max. |
| SNF_data.csv | **√** | **√** | **√** | **√** | The integrated results (similarity) matrix of SNF. |
| SNF_nogoldARIRes.csv | **√** | **√** | **√** | **√** | The ARI value between every two k of SNF results. |
| SNF_nogoldNMIRes.csv | **√** | **√** | **√** | **√** | The NMI value between every two k of SNF results. |
| SNF_nogoldRIRes.csv | **√** | **√** | **√** | **√** | The RI value between every two k of SNF results. |
| timeList.csv | **√** | **√** | **√** | **√** | The time consumption data. |
| timePie.svg | **√** | **√** | **√** | **√** | The figure of time consumption comparison. |
| ARI.RData |  | **√** |  | **√** | The ARI values comparing to the true labels of all methods’ results for each k. |
| F_SNF_2_heatmap.RData | **√** | **√** | **√** | **√** | The heatmap data of SNF result for k=2. This file will be generated in the situation of k=2 to *k*-max. |
| F_SNF_2_KM.RData | **√** | **√** | **√** | **√** | The KM data of SNF result for k=2. This file will be generated in the situation of k=2 to *k*-max. |
| NMI.RData |  | **√** |  | **√** | The NMI values comparing to the true labels of all methods’ results for each k. |
| nogold_method.RData | **√** | **√** | **√** | **√** | The RI, ARI and NMI value between every two methods of each k. |
| nogoldARIRes.RData | **√** | **√** | **√** | **√** | The ARI value between every two k of each method. |
| nogoldNMIRes.RData | **√** | **√** | **√** | **√** | The NMI value between every two k of each method. |
| nogoldRIRes.RData | **√** | **√** | **√** | **√** | The RI value between every two k of each method. |
| RI.RData |  | **√** |  | **√** | The RI values comparing to true labels of all methods’ results for each k. |
| sampleClu.RData | **√** | **√** | **√** | **√** | Consensus matrix which shows how many methods cluster each pair patients into the same cluster for each k. |
| SI.RData | **√** | **√** | **√** | **√** | The silhouette coefficient values of all methods’ results for each k. |
| timeList.RData | **√** | **√** | **√** | **√** | The time consumption data. |

*Note*: We take “SNF” as an example. S3* represents Scenario 3 with true labels. We notice that not all files in the table will be generated in all scenarios, and the tick represents this file will be generated in this scenario.

**Reference**

Nguyen, H., Shrestha, S., Draghici, S., and Nguyen, T. (2018). PINSPlus: a tool for tumor subtype discovery in integrated genomic data. *Bioinformatics*. doi:10.1093/bioinformatics/bty1049.

Sanchez-Vega, F., Mina, M., Armenia, J., Chatila, W. K., Luna, A., La, K. C., et al. (2018). Oncogenic Signaling Pathways in The Cancer Genome Atlas. Cell 173, 321-337.e10. doi:10.1016/j.cell.2018.03.035.
